# *Cynomorium songaricum* Extract Alleviates Memory Impairment through Increasing CREB/BDNF via Suppression of p38MAPK/ERK Pathway in Ovariectomized Rats

**DOI:** 10.1155/2019/9689325

**Published:** 2019-05-23

**Authors:** Fang-ze Tian, Hong-sheng Chang, Jian-xun Liu, Junchao Zheng, Dan Cheng, Yi Lu

**Affiliations:** ^1^Graduate School, Beijing University of Chinese Medicine, Beijing 100029, China; ^2^School of Preclinical Medicine, Beijing University of Chinese Medicines, Beijing, China; ^3^Xiyuan Hospital, China Academy of Chinese Medical Sciences, Haidian District, Beijing, China

## Abstract

*Cynomorium songaricum Rupr* is a very important traditional Chinese medicine for tonifying the kidney, which has a significant effect on improving estrogen level on the long term. In many studies, it can improve the learning and memory function of ovariectomized (OVX) model animals. 10 of the 50 rats received only bilateral back surgery and were harvested with the same amount of fat as the ovaries without removing the ovaries as sham group; remains underwent bilateral ovariectomy and equally randomized into five groups: sham group, with OVX as model group, estradiol valerate (EV, 0.2 mg/kg) as positive control, with 3.3 and 33 mg/kg body weight/day of ethyl acetate extract of* Cynomorium songaricum* extract (CSE) as low and high dosage groups, respectively. The orally administered CSE to ovariectomized rats exerted an ameliorative effect on learning and memory in the Morris water maze tests. All rats were sacrificed after 8 weeks of treatment, and tissue was analyzed using histopathology and electron microscopy. To comprehensively examine the mechanism, brain derived neurotrophic factor (BDNF), p-p38 mitogen-activated protein kinase (p-p38MAPK), extracellular regulated protein kinases (ERK), p-extracellular regulated protein kinases (p-ERK), and p-cAMP-response element binding protein (p-CREB) were detected by Western blotting. Using histopathology and electron microscopy, it was clearly observed that the pyramidal neurons of the hippocampal CA1 area were reduced in the OVX groups, indicating that CSE could attenuate the loss of pyramidal neurons in hippocampal CA1 and revert the synaptic morphological variations produced by ovariectomy. Mechanistically, the expressions of p-p38MAPK and p-ERK levels were significantly downregulated by CSE intervention, whereas the BDNF and p-CREB were significantly upregulated by CSE as compared to the control. Concisely,* Cynomorium songaricum Rupr* exhibited potential therapeutic effect on Neuroprotection of ovariectomized rats, and its effect was possibly exerted by p-CREB/BDNF mediated down regulation of ERK/p38MAPK.

## 1. Introduction

Clinical studies have demonstrated that estrogen can protect neurons [1]. Thus, estrogen treatment could be used to attenuate the prevalence of Alzheimer's disease (AD) and ameliorate learning and memory deficits [2, 3]. The cognitive dysfunction due to natural or surgical menopause is linked to the deficiency of the hormone estrogen. In general, experimental studies assessing the influence of estrogen on cognition are performed using ovariectomized (OVX) rats or mice. Menopausal hormone therapy, also called hormone replacement therapy (HRT), has been found to be effective in protecting against memory and learning dysfunction due to estrogen deficiency [4, 5]. However, currently used estrogenic treatments have several undesired effects, such as breast cancer and cardiac disorders [6, 7]. These findings argue for the need for further studies to explore new estrogenic treatments. Complementary and alternative medicines are recognized to have fewer undesired effects as well as promising therapeutic effectiveness against many chronic diseases, such as cognitive dysfunction.

Some laboratory discovered that phosphorylation of the extracellular signal-regulated kinase (ERK) was necessary for estradiol (E2) to enhance object recognition memory in ovariectomized female mice [8, 9]. They have since extended this finding to object placement (spatial memory) as well [10–13]. These findings demonstrated that the memory-enhancing effects of E2 depended on phosphorylation (i.e., activation) of a cell-signaling kinase. There is some precedence for sex differences in the mechanisms through which E2 regulates hippocampal function. In a study of neonatal rat hippocampal cultures, E2 interacted with mGluRs to increase ERK-dependent phosphorylation of cAMP response element binding (CREB) protein in females, but this phenomenon did not occur in males [14]. E2 enhances the memory consolidation of adult women as an important cause of mGluR activation [9], but E2 does not stimulate ERK-dependent CREB activation in male neonates, suggesting that E2-induced ERK activation is a gender difference. Subsequent studies have shown that E2 can increase the acetylation of the specific promoter H3 of the BDNF gene within 30 min. [12], a neurotrophin that is essential for hippocampal memory formation [15–17].

**Figure 1:**
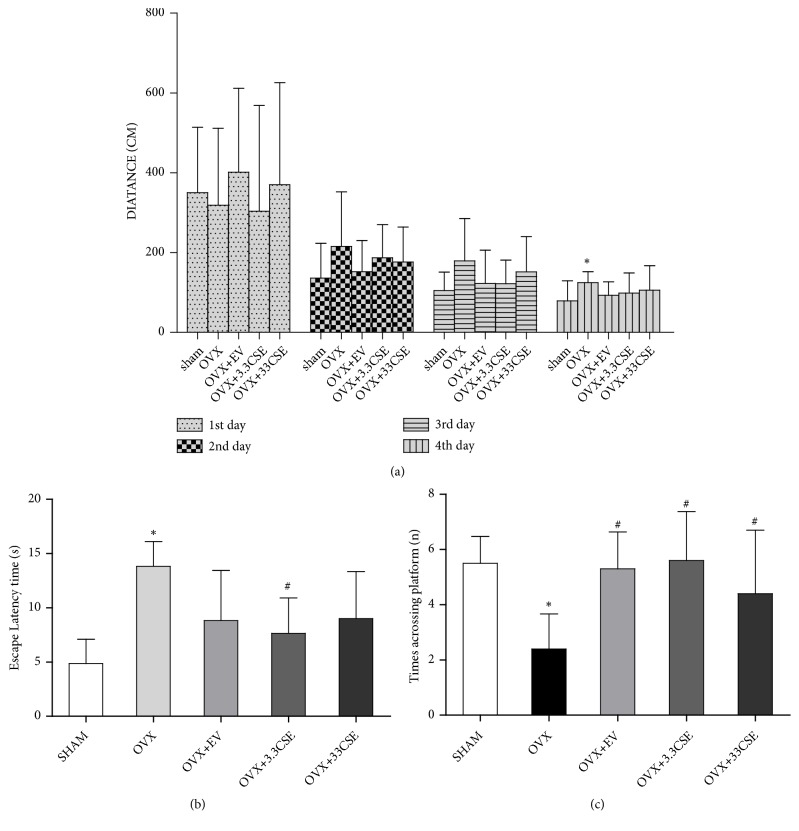
Effect of CSE on recognition memory in Morris water maze test. (a) The graph represents the escape latent distance in period of directional navigation of different groups. (b)-(c) Graph describes the escape latency and the times across platform in space probe test. Data were expressed as means ± SD (n = 10). ^*∗*^*P* < 0.05 vs. sham group, ^#^*P*<0.05 vs. OVX group.

Cynomorium is an important tonic Chinese medicine. This herb is extensively distributed in Asian hilly areas, especially in China and Mongolia.* Cynomorium songaricum* is useful against symptoms of aging, ranging from mild forms of memory impairment to dementia, regulating endocrinopathy, and improving sexual function [11, 18, 19]. Moreover, pharmacological studies have revealed that various ingredients obtained from this herb have a number of activities, such as anti-HCV protease [20], antiapoptosis, anticancer [21], antiprostatic hyperplasia [22], and fertility-promoting, actions [23–25].* Cynomorium songaricum* contains various active constituents including flavonoid, organic acids, and polysaccharides (Figure 1) [26–28], making it an important antimemory impairment herbal medicine in China.

*Cynomorium songaricum *extract (CSE) improves learning and memory* in vitro*, as studied through the water maze test method [20]; however,* in vivo *confirmation of this activity has not yet been confirmed. Thus, the present study was carried out to investigate the influence of CSE on spatial recognition memory in ovariectomized rats.

## 2. Materials and Methods

### 2.1. Animals

Fifty female rats were obtained from the Experimental Animal Center, The Academy of Military Medical Sciences (SD) (Certificate number: SCXK [jun] 2007-004). Their weight ranged between 180 and 200 g. Rats were housed in a room at a relative humidity of approximately 55%, 22°C temperature, and 12 h light/dark cycles. This study was approved by the University Ethical Committee on Research Practice at Beijing University of Chinese Medicines and performed in accordance with approved standards of laboratory animal care and use in experiments.

### 2.2. Ovariectomy

Fourty rats were ovariectomized under anesthesia induced by intraperitoneal injection of 7% chloral hydrate (250 mg/kg). For ovarian excision, approximately 5 cm incisions were made bilaterally in the dorsal-ventral region from the most posterior point of the rib cage. After the ovaries were excised, ovarian blood vessels were ligated by sterile thread. Sham-operated rats remove equal amounts of adipose tissue around the ovary. Following surgery, the rats were allowed to rest for 3 days. Water was given ad libitum. After ovariectomy, rat vaginal smears were detected by Wright's staining. If the vaginal cell smear loses its periodic changes, the white blood cells mainly last for more than 5 days, indicating that the estrogen level of the rat is decreased and entering the estrus interval.

### 2.3. Animal Treatment

Fourty rats were ovariectomized; six rats were assigned to sham-operation and maintained for 8 weeks. Rats were assigned to five experimental groups randomly with six animals for each group: the sham-operated (sham), ovariectomized (OVX, model), OVX + estradiol valerate (EV) treatment group, OVX + 3.3 CSE treatment group, and 33 CSE treatment group. The CSE and EV groups were treated intragastrically with CSE (33 mg/kg, 3.3mg/kg) and EV (0.2 mg/kg, Bayer, Germany) for 8 consecutive weeks starting on the third day after surgery, while rats in the sham and model groups were treated with distilled water only.

### 2.4. Preparation of* Cynomorium songaricum* Extract

*Cynomorium songaricum *Rupr was procured from the Anguo Chinese herbs market in Hebei province, People's Republic of China, authenticated as the fleshy stems by Professor Chun-Sheng Liu in Beijing University of Chinese Medicine, and the specimen was placed in university herbarium with voucher number CSE 601003042. For the preparation of CSE extract, the dried stems were crushed into small pieces and extracted using 20 kg powder with 8 volumes of 70% ethanol three times (each time for 1.5 h), followed by extraction with petroleum ether and ethyl acetate. CSE was dried at 60°C and stored at room temperature.

### 2.5. Morris Water Maze Test

The Morris water maze (MWM) test was used to assess spatial learning and memory of the rats. The test was conducted on day 7 after the start of drug administration. The MWM pool with a diameter of 120 cm was filled with water (22-25°C) and divided into four quadrants. An unseen platform was placed in the middle of the marked quadrant 1.5-2.0 cm under the surface of water. MWM consisted of two phases, the directional navigation period and the spatial probe test. In the former, all rats were given training 3 times per day for 4 days. First, rats were placed in platform-free quadrants. The trial ended when the rat reached the platform or after a maximum of one minute. If the rats reached the platform in less than one minute, that time was considered the escape latency. The escape latency was considered sixty seconds if the mouse could not find the platform within one minute. Then, the spatial probe test was carried out. For this test the platform was removed and the rat was permitted to swim for sixty seconds. The distance in the target area was determined. The Top View Animal Behavior Analyzing System (developed by the Institute of Materia Medica Chinese Academy of Medical Sciences, Beijing, China) was utilized to record and analyze data.

### 2.6. Morphometric Analysis

For morphometric analysis (Williams and Carter, 2009), 6 randomly selected rats from each group were anesthetized with 7% chloral hydrate and perfused with PBS (phosphate buffer saline, 0.1 M, 4°C) and then with 4% paraformaldehyde through the ascending aorta until stiffening of the tail and limbs. Subsequently, the brains were removed and divided into two parts: one part was stored in 4% paraformaldehyde for 7 days and sliced into coronal sections of 4 *μ*m thickness for hematoxylin and eosin (HE) staining, while the other part was used for fixing with 2.5% glutaraldehyde for electron microscopy.

### 2.7. Cell Culture

Neuro-2a cells were procured from the Cell Center, Peking Union Medical College. The culturing of these cells was carried out in 10 cm cell culture plates. The initial density was set at 5 × 10^4^ cells/ml. Containing 10% bovine serum albumin was used to culture the cells. The cells were used 36 h or 48 h after cell passage.

### 2.8. Cell Viability Analysis (CCK-8 Assay)

The cell viability (Studler et al., 1982) was assessed using a CCK-8 Cell Counting Kit-8. After treating cells with sequential concentrations of CSE (100 mg/L) for 22 h, 200 *μ*M H_2_O_2_ was added to each well for 2 h. Finally, the absorption at 450 nm was measured by a microplate reader (Thermo Labsystems, Helsinki, Finland).

### 2.9. Western Blot

#### 2.9.1. Animals

After the MWM test, three rats from each group were sacrificed for Western blot analysis to collect the hippocampal tissue. The tissue was homogenized in RIPA lysis buffer (1:5, w/v). Protein quantification was performed using a BCA protein assay kit. Accurately weighed 27 *μ*g of protein was used for electrophoresis. The proteins were transferred to polyvinylidene fluoride (PVDF) membranes, which were blocked with 5% fat-free milk powder in TBST buffer for a duration of one hour at room temperature, followed by incubation with primary antibody: p-p38 mitogen-activated protein kinase (1:1000), extracellular signal-regulated kinases (ERK) (1:1,000), brain-derived neurotrophic factor (BDNF) (1:2,000), p-cAMP-response element binding protein (p-CREB) (1:1,000), and p-extracellular regulated protein kinase (p-ERK) (1:2,000), acquired from Abcam, USA, for 12 h at 4°C. Subsequently, incubation of HRP-labeled goat anti-rabbit IgG (Jackson 111-035-003, 1:10,000) with the membrane was conducted for 60 minutes at room temperature. Finally, the visualization and scanning of the blots were carried out utilizing enhanced chemiluminescence (ECL) Western blot detection (Millipore WBKLS0500).

#### 2.9.2. Cells

Neuro-2a cells were washed with ice-cold PBS three times and harvested by adding ice-cold cell lysis buffer supplemented with PMSF. The protein was quantified using a BCA protein assay kit. Then, 40 *μ*g of protein was added to an 8% SDS-PAGE gel and transferred onto PVDF membranes. The primary antibodies p-p38 (1:1000), PSD95 (1:2000), and actin (1:2500), acquired from Abcam, USA, were incubated at 4°C overnight. The secondary antibody was incubated for 90 minutes. The bands were visualized using an ECL system (C600, Azure, USA).

Neuro-2a cells were treated with 100 mg / L CSE or 100 mg / L SB203580 for 22 hours each; 200 *μ*M H_2_O_2_ was added and then incubated for 2 hours. Then, the cells were lysed, and Western blot analysis was carried out using antibodies PSD95 and p-p38 to detect the effect of CSE.

## 3. Results

### 3.1. Effect of CSE on Spatial Recognition Memory

To evaluate cognitive function, spatial learning, and memory abilities, the MWM test was performed (Figure 1). The OVX rats did not differ from the sham group in terms of escape latency to find the platform on days 1-2 of training (directional navigation period). However, the latency distance to find the platform on days 3-5 of training was longer in OVX rats than in sham groups. Compared with OVX rats, the EV and CSE rats had a significantly lower escape latency distance (P < 0.05) in the navigation test.

Additional analyses were conducted to determine the escape latency and traversing time after the platform was removed from the maze (Figures 1(b) and 1(c)). After 4 days of treatment, compared with OVX rats, the CSE rats had decreased escape latencies and spent significantly more time traversing the position of the virtual platform (P < 0.05). These results suggest that there was obvious impairment of cognitive function and spatial learning and memory ability in the ovariectomized rats.

### 3.2. Morphological Changes of Hippocampus Tissues

#### 3.2.1. HE Staining

**Figure 2:**
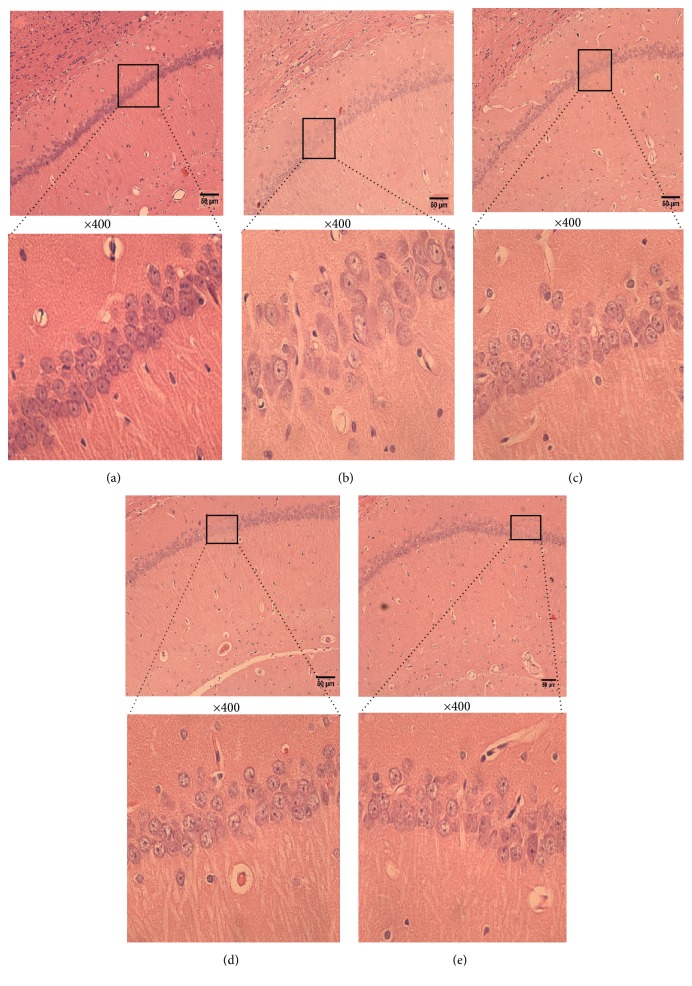
HE stains of hippocampal CA1 of brain after 8 w of OVX. (a) Sham group; (b) OVX group; (c) 0.2 mg/kg EV; (d) 3.3 mg/kg CSE. (e) 33 mg/kg CSE 100, 400× magnification. Scale bar: 50 *μ*m.

Histological changes in the neurons of the hippocampal CA1 region were obtained by HE staining in all groups (Figure 2). In the sham group, no histopathological abnormalities were observed (Figure 2(a)). However, most neurons in the CA1 region in the OVX group (Figure 2(b)) appeared to have triangulated pycnotic nuclei. Moreover, the image showed that the number of neurons was not affected in the sham group but decreased in the OVX group, while CSE treatment (Figures 2(d) and 2(e)) significantly increased the number of necrotic neurons.

#### 3.2.2. Transmission Electron Microscopy (TEM)

**Figure 3:**
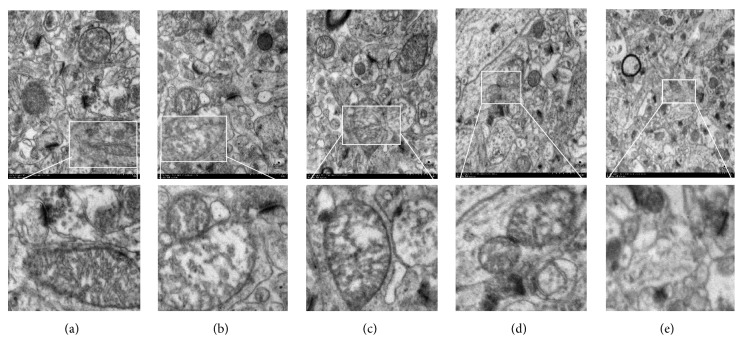
TEM of hippocampal CA1 of brain after 8 w of OVX. (a) Sham group; (b) OVX group; (c) 0.2 mg/kg EV; (d) 3.3 mg/kg CSE. (e) 33 mg/kg CSE, 6000×magnification. Scale bar: 200 *μ*m.

Under TEM, the pyramidal neurons of the hippocampal CA1 area in the sham group exhibited a clear nuclear membrane, evident nucleolus, rich organelles, and complete synaptic structure (Figure 3). The rats in the OVX group showed distorted nuclei with partial disappearance of decreased presynaptic vesicles and nuclear membrane damage (Figure 3(b)). After 8 weeks of treatment with CSE, there was a similarity in neural cells and synaptic structure of the CSE and sham groups (Figures 3(d) and 3(e)).

### 3.3. Effect of CES on the Protein Expressions of BDNF, p-p38, and p-CREB, ERK, and p-ERK in the Hippocampus

**Figure 4:**
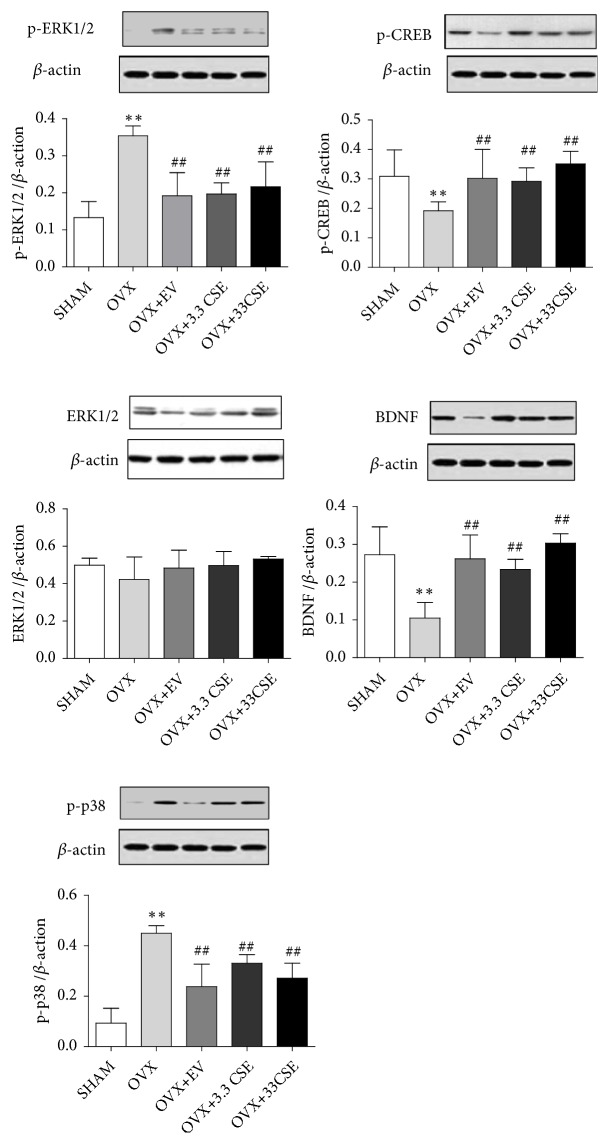
Western blot of hippocampal CA1 of brain after 8 w of OVX. (1) Sham group; (2) OVX group; (3) 0.2 mg/kg EV; (4) 3.3 mg/kg CSE. (5) 33mg/kg CSE. ^*∗∗*^*P* < 0.01 vs. sham group, ^##^*P*<0.01 vs. OVX group.

The results showed that CSE significantly (^##^*P* < 0.01) increased the expression of p-CREB and BDNF in the hippocampus of OVX rats and significantly (^##^*P *< 0.01) decreased the expression of p-p38 and p-ERK (^##^*P* < 0.01) (Figure 4).

### 3.4. CSE Attenuated Cell Loss Induced by H_*2*_O_*2*_ in Cultured Neuro-2a Cells

**Figure 5:**
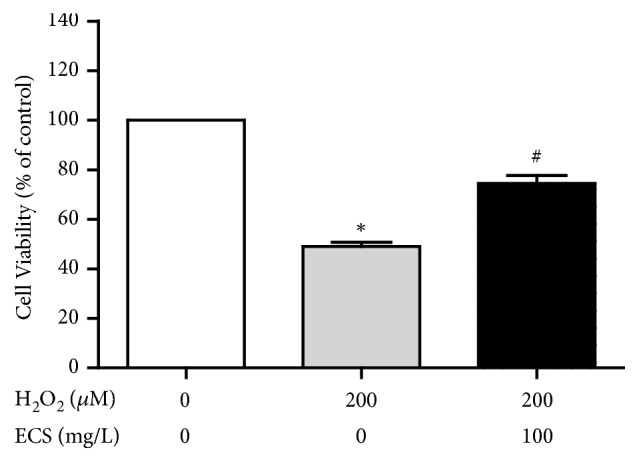
The effect of CSE on the viability of H_2_O_2_-induced Neuro 2a cells. (1) Sham group; (2) H_2_O_2_ group; (3) H_2_O_2_ + CSE group ^*∗*^*P* < 0.05 vs. control group, ^#^*P* < 0.05 vs. H_2_O_2_ group.

CSE was found to have protective effect against H_2_O_2_-induced damage of Neuro-2a cells, revealing the likely role of CSE against cognitive impairment (Figure 5).

### 3.5. p-p38 Pathway Was Involved in the Protection Effects of CSE

**Figure 6:**
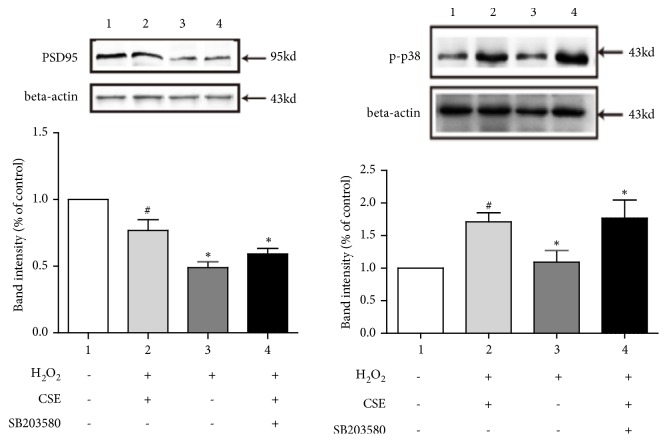
Western blot of PSD95 and p-p38 in H_2_O_2_-induced Neuro-2a cells. (1) Sham group; (2) H_2_O_2_ + CSE group; (3) H_2_O_2_ group; (4) H_2_O_2_ + CSE + SB203580 group. ^#^*P* < 0.05 vs. sham group, ^*∗*^*P*<0.05 vs. H_2_O_2_ group.

The results (Figure 6) showed that CSE had a protective effect against H_2_O_2_ damaged Neuro-2a cells, an improved expression of PSD95 compared with H_2_O_2_ group, and decreased expression of p-p38, indicating the alleviated cognition.

## 4. Discussion

The effects of estrogen on learning and memory are mainly dependent on the hippocampus and related nervous systems [29, 30]. Estrogen deficiency can cause a series of brain pathological changes such as memory, synaptic plasticity and neuronal morphology [31]. Estrogen replacement therapy can alleviate cognitive deficits caused by decreased estrogen after menopause, but this therapy does not produce the expected results in reversing memory loss in older women. For example, treatment with conjugated equine estrogens does not maintain or improve cognitive function in postmenopausal women over age 65, and in fact it can be detrimental to cognitive function in this population [32, 33]. In addition, hormone replacement therapy has a small risk of developing breast cancer, heart disease, and stroke but is statistically significant [34, 35]. Traditional Chinese medicines (TCM), such as CSE, provide a new approach to treat or prevent cognitive decline and related diseases.

Interestingly, the biochemical mechanisms underlying the memory-enhancing effects of E2 may differ between the sexes [36]. The ability of E2 to enhance object recognition and object placement memory consolidation in women depends on phosphorylation of ERK in the dorsal hippocampus [8, 10]. For example, in hippocampal cultures from neonatal rats, E2 interacts with mGluRs to increase ERK-dependent phosphorylation of cAMP response element binding (CREB) protein in females, but not in males [14].

Previous studies in our laboratory have shown that CSE has a good antioxidant effect [36] against SK-N-SH and PC12 cell injury triggered by hypoxanthine/xanthine oxidase (HPX/XO) or A*β*25-35t [37, 38]. More importantly, it proves that CSE has an estrogen-like effect. CSE plays a role of estrogen-like compounds and improved learning and memory through the MAPK pathway by combining with GPR30 [39]. In the current research, we aimed to investigate the mechanism of CSE to protect OVX rats.

The MWM test employed in the present study is a hippocampus-dependent memory task. This test is commonly employed in the evaluation of cognitive status [40]. The directional navigation training trials were utilized to explore spatial or place learning and the probe trials to examine whether the animal remembers the location of the platform, which indicates memory [41]. The latency time in the MWM was significantly shorter in the OVX rats after oral treatment with CSE. At the same time, we found that the 3.3mg/kg CSE treatment group is more capable of improving learning and memory in OVX rats.

The majority of studies have shown that hippocampus-dependent learning and memory are impaired by ovariectomy [42]. This study involved the attenuation of memory function in the OVX rats in the step-through passive avoidance test, and it was demonstrated that CSE improved this memory activity. The present results are consistent with previous findings showing that CSE plays a crucial role in the prevention or improvement of the cognitive loss triggered by OVX or D-galactose [43]. In short, the enhancement of memory activity by CSE was significant in the OVX rats.

The hippocampus is a crucial structure linked with learning and memory in humans and rodents [44]. In addition, the hippocampal CA1 region is very prone to degeneration [45]. Thus, the effects of CSE on cognitive processes in the hippocampal region were observed. In the present study, the potential mechanisms by which CSE enhanced learning and memory abilities were analyzed. The results showed that CSE significantly enhanced the number of CA1 pyramidal neurons in hippocampal slices obtained from OVX rats. CSE was found to have an ameliorating effect on neuronal cell viability and a neuroprotective influence on the hippocampal part of the OVX rats. The rats treated with CSE also exhibited an improved cell density compared to that of the OVX group. These results are in agreement with the findings of the behavioral analysis in the present study that the EV group had improved learning and memory function. However, the exact mode of action by which CSE improves the abilities of learning and memory in non-OVX rats is unclear.

Published work indicates that E2 enhances memory consolidation in ovariectomized female mice by rapidly activating ERK via ER*α*/*β*-mGluR1a interactions, NMDA receptors, and activation of PI3K/Akt and PKA [10, 46, 47]. ERK phosphorylation triggers activation of mTOR signaling and CA1 dendritic spinogenesis [46, 48], as well as histone H3 acetylation of BDNF and transcription of multiple other genes. The p38MAPK pathway plays an important role in cell proliferation and differentiation; that is, the inhibition of the p38MAPK pathway reduces the production of inflammatory mediators and inhibits apoptosis, so p38 inhibitors are being used in the development of antineoplastic drugs [49]. The present study revealed an increase in p38MAPK protein expression in OVX rats; thus, the pathological effects of the hippocampal CA1 subfield in the sham and OVX groups were significantly different. CSE significantly reduced the phosphorylation level of p38MAPK, leading to the restricted apoptotic effect of CSE. This result indicates that CSE inhibits OVX- induced p38 phosphorylation by GPR30.

Evidence-based studies have shown that estrogen receptors can influence phosphorylated CREB (p-CREB) to regulate synaptic plasticity in the hippocampal CA1 region of adult rats. Neuronal survival and plasticity were assessed by measuring expression of p-CREB and the level of its downstream factor BDNF [50–52]. In this study, an increase in the expression of p-CREB and BDNF proteins was observed in CSE-supplemented OVX rats.

These findings together revealed that the improved synaptic plasticity and cognition are closely associated with the ameliorated expression of p-CREB and BDNF. Therefore, BDNF could be involved in CREB-provoked regulation of synaptic plasticity and cognitive activity with CSE treatment. These findings are valuable for supporting the assumption that CSE influences cognition and synaptic plasticity in OVX rats.

### 4.1. Statistical Analysis

Graph Pad Prism 5 (GraphPad Software Incorporated, La Jolla, CA) was used to construct graphs. Data analysis was carried out using one-way ANOVA or Student's t-test utilizing Statistical Package for Social Sciences (SPSS) 13.0. All data were narrated as mean±standard error, while the statistical significance of difference was determined by P < 0.05.

## Data Availability

The data used to support the findings of this study are available from the corresponding author upon request.
